# Dichloridobis[(1*S*,1*S*′,2*R*,2*R*′)-(+)-1,1′-di-*tert*-butyl-2,2′-diphospho­lane-κ^2^ *P*,*P*′]ruthenium(II)

**DOI:** 10.1107/S1600536808008301

**Published:** 2008-05-03

**Authors:** Chubei Wang, Haiyan Tao, Baoming Ji

**Affiliations:** a College of Chemistry, Central China Normal University, Wuhan, Hubei 430072, People’s Republic of China; b College of Chemistry and Molecular Science, Wuhan University, Wuhan, Hubei 430072, People’s Republic of China; c College of Chemistry and Chemical Engineering, Luoyang Normal University, Luoyang, Henan 471022, People’s Republic of China

## Abstract

In the title compound, [RuCl_2_(C_16_H_32_P_2_)_2_], the Ru^II^ ion is situated on a twofold rotation axis, so the asymmetric unit contains one half-mol­ecule. The slightly distorted octa­hedral environment of the Ru center is formed by four P atoms [Ru—P = 2.4417 (6) and 2.4544 (6) Å] from two different (1*S*,1*S*′,2*R*,2*R*′)-TangPhos ligands [(1*S*,1*S*′,2*R*,2*R*′)-TangPhos = (1*S*,1*S*′,2*R*,2*R*′)-(+)-1,1′-di-*tert*-butyl-2,2′-diphospho­lane] and two Cl atoms [Ru—Cl = 2.4267 (5) Å].

## Related literature

For related literature, see: Ikariya *et al.* (1985 ▶); James & Fogg (1993 ▶); Stoop *et al.* (1999 ▶).
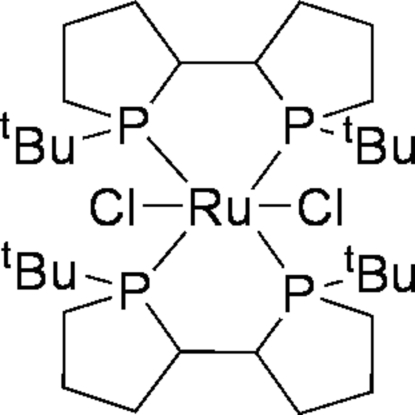


## Experimental

### 

#### Crystal data

[RuCl_2_(C_16_H_32_P_2_)_2_]
*M* *_r_* = 744.72
Orthorhombic, 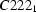

*a* = 11.8640 (14) Å
*b* = 20.669 (3) Å
*c* = 14.2274 (17) Å
*V* = 3488.8 (8) Å^3^
*Z* = 4
Mo *K*α radiation
μ = 0.81 mm^−1^
*T* = 108 (2) K
0.24 × 0.15 × 0.10 mm

#### Data collection

Bruker SMART CCD area-detector diffractometer
Absorption correction: multi-scan (*SADABS*; Bruker, 2001 ▶) *T*_min_ = 0.863, *T*_max_ = 0.921
11542 measured reflections
4164 independent reflections
4018 reflections with *I* > 2σ(*I*)
*R*_int_ = 0.025

#### Refinement

*R*[*F* ^2^ > 2σ(*F* ^2^)] = 0.024
*wR*(*F* ^2^) = 0.057
*S* = 1.06
4164 reflections
183 parameters
H-atom parameters not refined
Δρ_max_ = 0.83 e Å^−3^
Δρ_min_ = −0.29 e Å^−3^
Absolute structure: Flack (1983 ▶), 1750 Friedel pairs
Flack parameter: 0.00 (2)

### 

Data collection: *SMART* (Bruker, 1997 ▶); cell refinement: *SAINT* (Bruker, 2001 ▶); data reduction: *SAINT*; program(s) used to solve structure: *SHELXS97* (Sheldrick, 2008 ▶); program(s) used to refine structure: *SHELXL97* (Sheldrick, 2008 ▶); molecular graphics: *SHELXTL* (Sheldrick, 2008 ▶); software used to prepare material for publication: *SHELXTL*.

## Supplementary Material

Crystal structure: contains datablocks global, I. DOI: 10.1107/S1600536808008301/cv2390sup1.cif

Structure factors: contains datablocks I. DOI: 10.1107/S1600536808008301/cv2390Isup2.hkl

Additional supplementary materials:  crystallographic information; 3D view; checkCIF report

## Figures and Tables

**Fig. 1.**
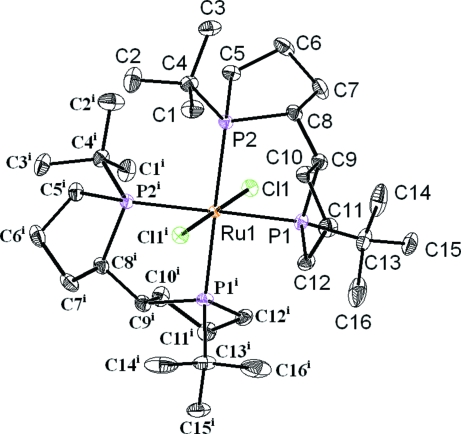
View of the title compound with the atomic numbering and 40% probability displacement ellipsoids [symmetry code: (i) -x, y, -z + 1/2]. H atoms are omitted for clarity.

## supplementary crystallographic information

### Comment

Recently, some chiral diphosphino ruthenium complexes have been synthesized and used as catalysts for the asymmetric reactions (Stoop *et al.*, 1999; James *et al.*, 1993). Herein, we report the synthesis and crystal structure of the title compound - the ruthenium(II) complex containing the chiral TangPhos ligand.

As shown in Fig. 1, the crystals of the title complex contain discrete [RuCl_2_(TangPhos)_2_] units with the metal center in a slightly distorted octahedral environment. The *trans*-axial positions of Ru^II^ environment are occupied by Cl1 and Cl1^i^ atoms, and the equatorial positions are occupied by P1, P2, P1^i^, P2^i^ atoms, respectively [symmetry code: (i) -*x*, *y*, -*z* + 1/2], from two different TangPhos ligands, resulting in two chelate rings, which assume a half-chair conformation with the *tert*-butyl in the less hindered equatorial positions. Similar conformations were found in previously reported related structures (Stoop *et al.*, 1999; Ikariya *et al.*, 1985).

### Experimental

To a solution of [RuCl_2_(PPh)_3_] (96 mg, 0.1 mmol) in 2 ml of deoxygenated CH_2_Cl_2_ was added dropwise a solution of (1*S*,1S',2*R*,2*R*')-TangPhos, (56 mg, 0.2 mmol) in CH_2_Cl_2_ (1 ml). The resulting mixture was stirred at ambient temperature for 6 h. Deoxygenated ether (12 ml) was added into the vigorously stirring solution and kept for 6 h at room temperature. The resulting light brown precipitate was filtered, washed with ether (3 times with 10 ml), and dried under vacuum. Yield: 45 mg (56%). Crystals suitable for X-ray diffraction were obtained by diffusion of hexane into a CD_2_Cl_2_ solution of the above compound at room temperature.

### Refinement

All H atoms were positioned geometrically (C—H 0.98–1.00 Å), and treated as riding, with *U*_iso_(H) = 1.2–1.5*U*_eq_ (C).

### Crystal data

**Table:**

[RuCl_2_(C_16_H_32_P_2_)_2_]	*F*_000_ = 1576
*M**_r_* = 744.72	*D*_x_ = 1.418 Mg m^−^^3^
Orthorhombic, *C*222_1_	Mo *K*α radiation λ = 0.71073 Å
Hall symbol: C 2c 2	Cell parameters from 5992 reflections
*a* = 11.8640 (14) Å	θ = 2.4–28.2º
*b* = 20.669 (3) Å	µ = 0.81 mm^−^^1^
*c* = 14.2274 (17) Å	*T* = 108 (2) K
*V* = 3488.8 (8) Å^3^	Brick, orange
*Z* = 4	0.24 × 0.15 × 0.10 mm

### Data collection

**Table:**

Bruker SMART CCD area-detector diffractometer	4164 independent reflections
Radiation source: fine-focus sealed tube	4018 reflections with *I* > 2σ(*I*)
Monochromator: graphite	*R*_int_ = 0.025
*T* = 108(2) K	θ_max_ = 28.3º
phi and ω scans	θ_min_ = 2.0º
Absorption correction: multi-scan(SADABS; Bruker, 2001)	*h* = −14→15
*T*_min_ = 0.863, *T*_max_ = 0.921	*k* = −17→27
11542 measured reflections	*l* = −18→18

### Refinement

**Table:**

Refinement on *F*^2^	Hydrogen site location: inferred from neighbouring sites
Least-squares matrix: full	H-atom parameters not refined
*R*[*F*^2^ > 2σ(*F*^2^)] = 0.024	*w* = 1/[σ^2^(*F*_o_^2^) + (0.0315*P*)^2^ + 0.2939*P*] where *P* = (*F*_o_^2^ + 2*F*_c_^2^)/3
*wR*(*F*^2^) = 0.057	(Δ/σ)_max_ = 0.003
*S* = 1.06	Δρ_max_ = 0.83 e Å^−^^3^
4164 reflections	Δρ_min_ = −0.29 e Å^−^^3^
183 parameters	Extinction correction: none
Primary atom site location: structure-invariant direct methods	Absolute structure: Flack (1983), 1750 Friedel pairs
Secondary atom site location: difference Fourier map	Flack parameter: 0.00 (2)

### Special details

**Table:**

Geometry. All e.s.d.'s (except the e.s.d. in the dihedral angle between two l.s. planes) are estimated using the full covariance matrix. The cell e.s.d.'s are taken into account individually in the estimation of e.s.d.'s in distances, angles and torsion angles; correlations between e.s.d.'s in cell parameters are only used when they are defined by crystal symmetry. An approximate (isotropic) treatment of cell e.s.d.'s is used for estimating e.s.d.'s involving l.s. planes.
Refinement. Refinement of *F*^2^ against ALL reflections. The weighted *R*-factor *wR* and goodness of fit *S* are based on *F*^2^, conventional *R*-factors *R* are based on *F*, with *F* set to zero for negative *F*^2^. The threshold expression of *F*^2^ > σ(*F*^2^) is used only for calculating *R*-factors(gt) *etc*. and is not relevant to the choice of reflections for refinement. *R*-factors based on *F*^2^ are statistically about twice as large as those based on *F*, and *R*- factors based on ALL data will be even larger.

### Fractional atomic coordinates and isotropic or equivalent isotropic displacement parameters (Å^2^)

**Table:**

	*x*	*y*	*z*	*U*_iso_*/*U*_eq_	
C1	0.02414 (19)	0.16346 (11)	−0.00952 (15)	0.0256 (5)	
H1A	−0.0059	0.1290	−0.0497	0.038*	
H1B	0.0806	0.1883	−0.0445	0.038*	
H1C	−0.0373	0.1922	0.0098	0.038*	
C2	−0.0038 (3)	0.08613 (11)	0.12059 (16)	0.0353 (6)	
H2A	−0.0740	0.1086	0.1364	0.053*	
H2B	0.0291	0.0677	0.1778	0.053*	
H2C	−0.0198	0.0514	0.0756	0.053*	
C3	0.1820 (2)	0.09489 (11)	0.04344 (16)	0.0257 (5)	
H3A	0.2177	0.0737	0.0973	0.039*	
H3B	0.2361	0.1243	0.0136	0.039*	
H3C	0.1580	0.0621	−0.0021	0.039*	
C4	0.07852 (18)	0.13369 (11)	0.07717 (15)	0.0188 (4)	
C5	0.21860 (15)	0.15194 (10)	0.24348 (17)	0.0185 (4)	
H5A	0.2097	0.1649	0.3100	0.022*	
H5B	0.2031	0.1050	0.2384	0.022*	
C6	0.33823 (17)	0.16652 (12)	0.21003 (18)	0.0262 (5)	
H6A	0.3927	0.1616	0.2622	0.031*	
H6B	0.3601	0.1369	0.1584	0.031*	
C7	0.33514 (17)	0.23639 (12)	0.17560 (17)	0.0261 (5)	
H7A	0.4066	0.2474	0.1434	0.031*	
H7B	0.3250	0.2663	0.2293	0.031*	
C8	0.23545 (18)	0.24229 (10)	0.10707 (15)	0.0179 (4)	
H8	0.2549	0.2176	0.0489	0.021*	
C9	0.20454 (18)	0.31083 (11)	0.07802 (15)	0.0185 (4)	
H9	0.2760	0.3358	0.0695	0.022*	
C10	0.13653 (19)	0.31551 (11)	−0.01378 (14)	0.0220 (5)	
H10A	0.1839	0.3027	−0.0680	0.026*	
H10B	0.0702	0.2865	−0.0112	0.026*	
C11	0.09906 (19)	0.38550 (11)	−0.02376 (15)	0.0237 (5)	
H11A	0.0465	0.3904	−0.0773	0.028*	
H11B	0.1647	0.4142	−0.0338	0.028*	
C12	0.04012 (19)	0.40157 (10)	0.06914 (15)	0.0203 (5)	
H12A	−0.0402	0.3887	0.0663	0.024*	
H12B	0.0442	0.4486	0.0817	0.024*	
C13	0.2153 (2)	0.42001 (11)	0.21194 (17)	0.0261 (5)	
C14	0.3123 (2)	0.39022 (14)	0.26745 (19)	0.0414 (7)	
H14A	0.3582	0.4247	0.2951	0.062*	
H14B	0.3590	0.3640	0.2253	0.062*	
H14C	0.2819	0.3628	0.3176	0.062*	
C15	0.2678 (2)	0.46048 (11)	0.13264 (17)	0.0291 (5)	
H15A	0.2078	0.4806	0.0954	0.044*	
H15B	0.3134	0.4324	0.0921	0.044*	
H15C	0.3158	0.4943	0.1598	0.044*	
C16	0.1502 (3)	0.46594 (13)	0.2754 (2)	0.0505 (9)	
H16A	0.1180	0.4417	0.3281	0.076*	
H16B	0.0895	0.4864	0.2394	0.076*	
H16C	0.2013	0.4993	0.2995	0.076*	
Cl1	−0.13351 (4)	0.27771 (3)	0.12078 (3)	0.01700 (10)	
P1	0.11419 (5)	0.35562 (3)	0.16379 (4)	0.01581 (11)	
P2	0.11916 (4)	0.19819 (3)	0.16823 (4)	0.01347 (11)	
Ru1	0.0000	0.277275 (10)	0.2500	0.01108 (6)	

### Atomic displacement parameters (Å^2^)

**Table:**

	*U*^11^	*U*^22^	*U*^33^	*U*^12^	*U*^13^	*U*^23^
C1	0.0294 (13)	0.0265 (11)	0.0207 (11)	0.0048 (10)	−0.0060 (9)	−0.0074 (9)
C2	0.0464 (14)	0.0316 (12)	0.0279 (12)	−0.0175 (14)	0.0162 (14)	−0.0125 (10)
C3	0.0352 (13)	0.0209 (11)	0.0209 (11)	0.0099 (10)	0.0033 (10)	−0.0011 (9)
C4	0.0241 (11)	0.0182 (10)	0.0142 (10)	0.0018 (9)	0.0029 (8)	−0.0020 (8)
C5	0.0201 (10)	0.0212 (9)	0.0141 (9)	0.0048 (8)	0.0021 (9)	0.0025 (10)
C6	0.0168 (11)	0.0359 (14)	0.0260 (11)	0.0088 (10)	0.0036 (9)	0.0107 (10)
C7	0.0132 (10)	0.0342 (15)	0.0310 (12)	−0.0006 (9)	0.0012 (9)	0.0117 (10)
C8	0.0166 (10)	0.0211 (11)	0.0159 (9)	0.0012 (8)	0.0046 (8)	0.0031 (8)
C9	0.0181 (10)	0.0208 (11)	0.0166 (10)	0.0011 (8)	0.0045 (8)	0.0041 (9)
C10	0.0285 (12)	0.0242 (12)	0.0133 (10)	0.0052 (10)	0.0058 (9)	0.0023 (8)
C11	0.0304 (13)	0.0240 (12)	0.0166 (11)	0.0015 (10)	0.0039 (9)	0.0061 (9)
C12	0.0247 (11)	0.0162 (10)	0.0199 (11)	0.0009 (9)	0.0015 (8)	0.0037 (9)
C13	0.0326 (13)	0.0224 (12)	0.0234 (11)	−0.0126 (10)	0.0024 (10)	−0.0004 (10)
C14	0.0485 (16)	0.0439 (15)	0.0317 (17)	−0.0285 (13)	−0.0166 (12)	0.0120 (12)
C15	0.0348 (13)	0.0212 (11)	0.0315 (13)	−0.0111 (11)	0.0056 (11)	0.0024 (10)
C16	0.0582 (19)	0.0429 (17)	0.050 (2)	−0.0304 (15)	0.0255 (15)	−0.0289 (14)
Cl1	0.0168 (2)	0.0205 (2)	0.0137 (2)	0.0003 (2)	−0.00355 (16)	0.0000 (2)
P1	0.0188 (3)	0.0144 (3)	0.0143 (3)	−0.0034 (2)	0.0013 (2)	0.0013 (2)
P2	0.0146 (3)	0.0147 (2)	0.0111 (2)	0.0011 (2)	0.0020 (2)	0.0013 (2)
Ru1	0.01224 (10)	0.01165 (10)	0.00935 (9)	0.000	0.00015 (8)	0.000

### Geometric parameters (Å, °)

**Table:**

C1—C4	1.522 (3)	C10—C11	1.520 (3)
C1—H1A	0.9800	C10—H10A	0.9900
C1—H1B	0.9800	C10—H10B	0.9900
C1—H1C	0.9800	C11—C12	1.532 (3)
C2—C4	1.517 (3)	C11—H11A	0.9900
C2—H2A	0.9800	C11—H11B	0.9900
C2—H2B	0.9800	C12—P1	1.868 (2)
C2—H2C	0.9800	C12—H12A	0.9900
C3—C4	1.543 (3)	C12—H12B	0.9900
C3—H3A	0.9800	C13—C16	1.520 (4)
C3—H3B	0.9800	C13—C14	1.525 (3)
C3—H3C	0.9800	C13—C15	1.537 (3)
C4—P2	1.920 (2)	C13—P1	1.918 (2)
C5—C6	1.527 (3)	C14—H14A	0.9800
C5—P2	1.858 (2)	C14—H14B	0.9800
C5—H5A	0.9900	C14—H14C	0.9800
C5—H5B	0.9900	C15—H15A	0.9800
C6—C7	1.525 (3)	C15—H15B	0.9800
C6—H6A	0.9900	C15—H15C	0.9800
C6—H6B	0.9900	C16—H16A	0.9800
C7—C8	1.538 (3)	C16—H16B	0.9800
C7—H7A	0.9900	C16—H16C	0.9800
C7—H7B	0.9900	Cl1—Ru1	2.4267 (5)
C8—C9	1.521 (3)	P1—Ru1	2.4417 (6)
C8—P2	1.869 (2)	P2—Ru1	2.4544 (6)
C8—H8	1.0000	Ru1—Cl1^i^	2.4267 (5)
C9—C10	1.538 (3)	Ru1—P1^i^	2.4417 (6)
C9—P1	1.870 (2)	Ru1—P2^i^	2.4544 (6)
C9—H9	1.0000		
			
C4—C1—H1A	109.5	C12—C11—H11A	110.7
C4—C1—H1B	109.5	C10—C11—H11B	110.7
H1A—C1—H1B	109.5	C12—C11—H11B	110.7
C4—C1—H1C	109.5	H11A—C11—H11B	108.8
H1A—C1—H1C	109.5	C11—C12—P1	107.29 (15)
H1B—C1—H1C	109.5	C11—C12—H12A	110.3
C4—C2—H2A	109.5	P1—C12—H12A	110.3
C4—C2—H2B	109.5	C11—C12—H12B	110.3
H2A—C2—H2B	109.5	P1—C12—H12B	110.3
C4—C2—H2C	109.5	H12A—C12—H12B	108.5
H2A—C2—H2C	109.5	C16—C13—C14	109.1 (2)
H2B—C2—H2C	109.5	C16—C13—C15	107.6 (2)
C4—C3—H3A	109.5	C14—C13—C15	107.1 (2)
C4—C3—H3B	109.5	C16—C13—P1	109.12 (17)
H3A—C3—H3B	109.5	C14—C13—P1	112.12 (17)
C4—C3—H3C	109.5	C15—C13—P1	111.66 (16)
H3A—C3—H3C	109.5	C13—C14—H14A	109.5
H3B—C3—H3C	109.5	C13—C14—H14B	109.5
C2—C4—C1	108.6 (2)	H14A—C14—H14B	109.5
C2—C4—C3	107.60 (19)	C13—C14—H14C	109.5
C1—C4—C3	107.18 (17)	H14A—C14—H14C	109.5
C2—C4—P2	109.67 (14)	H14B—C14—H14C	109.5
C1—C4—P2	111.87 (15)	C13—C15—H15A	109.5
C3—C4—P2	111.76 (15)	C13—C15—H15B	109.5
C6—C5—P2	108.00 (15)	H15A—C15—H15B	109.5
C6—C5—H5A	110.1	C13—C15—H15C	109.5
P2—C5—H5A	110.1	H15A—C15—H15C	109.5
C6—C5—H5B	110.1	H15B—C15—H15C	109.5
P2—C5—H5B	110.1	C13—C16—H16A	109.5
H5A—C5—H5B	108.4	C13—C16—H16B	109.5
C7—C6—C5	105.35 (17)	H16A—C16—H16B	109.5
C7—C6—H6A	110.7	C13—C16—H16C	109.5
C5—C6—H6A	110.7	H16A—C16—H16C	109.5
C7—C6—H6B	110.7	H16B—C16—H16C	109.5
C5—C6—H6B	110.7	C12—P1—C9	92.92 (10)
H6A—C6—H6B	108.8	C12—P1—C13	101.48 (10)
C6—C7—C8	107.28 (19)	C9—P1—C13	102.59 (10)
C6—C7—H7A	110.3	C12—P1—Ru1	116.00 (7)
C8—C7—H7A	110.3	C9—P1—Ru1	108.52 (7)
C6—C7—H7B	110.3	C13—P1—Ru1	128.89 (8)
C8—C7—H7B	110.3	C5—P2—C8	92.89 (9)
H7A—C7—H7B	108.5	C5—P2—C4	101.01 (10)
C9—C8—C7	115.57 (18)	C8—P2—C4	102.11 (9)
C9—C8—P2	113.76 (15)	C5—P2—Ru1	115.80 (8)
C7—C8—P2	103.53 (14)	C8—P2—Ru1	108.73 (7)
C9—C8—H8	107.9	C4—P2—Ru1	129.60 (7)
C7—C8—H8	107.9	Cl1^i^—Ru1—Cl1	179.58 (3)
P2—C8—H8	107.9	Cl1^i^—Ru1—P1	90.913 (19)
C8—C9—C10	114.56 (18)	Cl1—Ru1—P1	88.806 (19)
C8—C9—P1	114.98 (15)	Cl1^i^—Ru1—P1^i^	88.806 (19)
C10—C9—P1	102.85 (14)	Cl1—Ru1—P1^i^	90.913 (19)
C8—C9—H9	108.0	P1—Ru1—P1^i^	96.91 (3)
C10—C9—H9	108.0	Cl1^i^—Ru1—P2^i^	91.107 (19)
P1—C9—H9	108.0	Cl1—Ru1—P2^i^	89.175 (19)
C11—C10—C9	107.00 (18)	P1—Ru1—P2^i^	177.969 (19)
C11—C10—H10A	110.3	P1^i^—Ru1—P2^i^	83.341 (18)
C9—C10—H10A	110.3	Cl1^i^—Ru1—P2	89.175 (19)
C11—C10—H10B	110.3	Cl1—Ru1—P2	91.107 (19)
C9—C10—H10B	110.3	P1—Ru1—P2	83.341 (18)
H10A—C10—H10B	108.6	P1^i^—Ru1—P2	177.969 (19)
C10—C11—C12	105.02 (17)	P2^i^—Ru1—P2	96.48 (3)
C10—C11—H11A	110.7		
			
P2—C5—C6—C7	33.0 (2)	C9—C8—P2—C4	110.84 (16)
C5—C6—C7—C8	−50.9 (2)	C7—C8—P2—C4	−122.93 (15)
C6—C7—C8—C9	169.48 (18)	C9—C8—P2—Ru1	−28.74 (16)
C6—C7—C8—P2	44.4 (2)	C7—C8—P2—Ru1	97.50 (14)
C7—C8—C9—C10	159.29 (18)	C2—C4—P2—C5	76.02 (19)
P2—C8—C9—C10	−81.1 (2)	C1—C4—P2—C5	−163.42 (15)
C7—C8—C9—P1	−81.9 (2)	C3—C4—P2—C5	−43.22 (17)
P2—C8—C9—P1	37.76 (19)	C2—C4—P2—C8	171.43 (17)
C8—C9—C10—C11	171.88 (18)	C1—C4—P2—C8	−68.01 (17)
P1—C9—C10—C11	46.39 (19)	C3—C4—P2—C8	52.19 (17)
C9—C10—C11—C12	−53.0 (2)	C2—C4—P2—Ru1	−61.4 (2)
C10—C11—C12—P1	33.8 (2)	C1—C4—P2—Ru1	59.14 (18)
C11—C12—P1—C9	−6.50 (16)	C3—C4—P2—Ru1	179.34 (11)
C11—C12—P1—C13	96.98 (16)	C12—P1—Ru1—Cl1^i^	−160.02 (8)
C11—C12—P1—Ru1	−118.75 (14)	C9—P1—Ru1—Cl1^i^	97.09 (7)
C8—C9—P1—C12	−147.28 (16)	C13—P1—Ru1—Cl1^i^	−27.34 (10)
C10—C9—P1—C12	−22.07 (15)	C12—P1—Ru1—Cl1	19.66 (8)
C8—C9—P1—C13	110.27 (16)	C9—P1—Ru1—Cl1	−83.23 (7)
C10—C9—P1—C13	−124.52 (15)	C13—P1—Ru1—Cl1	152.34 (10)
C8—C9—P1—Ru1	−28.60 (16)	C12—P1—Ru1—P1^i^	−71.11 (8)
C10—C9—P1—Ru1	96.61 (13)	C9—P1—Ru1—P1^i^	−174.00 (8)
C16—C13—P1—C12	77.9 (2)	C13—P1—Ru1—P1^i^	61.56 (10)
C14—C13—P1—C12	−161.05 (17)	C12—P1—Ru1—P2	110.92 (8)
C15—C13—P1—C12	−40.84 (19)	C9—P1—Ru1—P2	8.03 (7)
C16—C13—P1—C9	173.58 (18)	C13—P1—Ru1—P2	−116.41 (10)
C14—C13—P1—C9	−65.40 (19)	C5—P2—Ru1—Cl1^i^	20.56 (7)
C15—C13—P1—C9	54.81 (19)	C8—P2—Ru1—Cl1^i^	−82.33 (7)
C16—C13—P1—Ru1	−59.7 (2)	C4—P2—Ru1—Cl1^i^	153.05 (9)
C14—C13—P1—Ru1	61.3 (2)	C5—P2—Ru1—Cl1	−159.76 (7)
C15—C13—P1—Ru1	−178.44 (13)	C8—P2—Ru1—Cl1	97.35 (7)
C6—C5—P2—C8	−6.72 (17)	C4—P2—Ru1—Cl1	−27.27 (9)
C6—C5—P2—C4	96.21 (17)	C8—P2—Ru1—P1	8.68 (7)
C6—C5—P2—Ru1	−119.15 (15)	C5—P2—Ru1—P2^i^	−70.46 (7)
C9—C8—P2—C5	−147.25 (16)	C8—P2—Ru1—P2^i^	−173.36 (8)
C7—C8—P2—C5	−21.02 (16)	C4—P2—Ru1—P2^i^	62.03 (9)

Symmetry codes: (i) −*x*, *y*, −*z*+1/2.
